# Peculiar Case of Scarlatina

**Published:** 1871-04

**Authors:** 


Peculiar Case of Scarlatina.—At a meeting of the Pathological Society of Dublin, held on the 4th inst., Dr. Henry Kennedy exhibited an entire clavicle which had come away from a child, aged three, attacked with scarlatina. It seems an abscess had formed in the lower part of the neck on the left side, and, as a consequence, the clavicle of that side was entirely removed. The case is, I believe, unique, as a similar termination, to my knowledge, has never been recorded. It may be mentioned that the child recovered, and is now in tolerably good health.—*Lancet, March 11th.*

